# Bayesian integration of position and orientation cues in perception of biological and non-biological forms

**DOI:** 10.3389/fnhum.2014.00091

**Published:** 2014-02-24

**Authors:** Steven M. Thurman, Hongjing Lu

**Affiliations:** ^1^Department of Psychology, University of California Los AngelesLos Angeles, CA, USA; ^2^Department of Statistics, University of California Los AngelesLos Angeles, CA, USA

**Keywords:** visual perception, Bayesian model, biological motion, sensory integration, cue reliability, form analysis

## Abstract

Visual form analysis is fundamental to shape perception and likely plays a central role in perception of more complex dynamic shapes, such as moving objects or biological motion. Two primary form-based cues serve to represent the overall shape of an object: the spatial position and the orientation of locations along the boundary of the object. However, it is unclear how the visual system integrates these two sources of information in dynamic form analysis, and in particular how the brain resolves ambiguities due to sensory uncertainty and/or cue conflict. In the current study, we created animations of sparsely-sampled dynamic objects (human walkers or rotating squares) comprised of oriented Gabor patches in which orientation could either coincide or conflict with information provided by position cues. When the cues were incongruent, we found a characteristic trade-off between position and orientation information whereby position cues increasingly dominated perception as the relative uncertainty of orientation increased and vice versa. Furthermore, we found no evidence for differences in the visual processing of biological and non-biological objects, casting doubt on the claim that biological motion may be specialized in the human brain, at least in specific terms of form analysis. To explain these behavioral results quantitatively, we adopt a probabilistic template-matching model that uses Bayesian inference within local modules to estimate object shape separately from either spatial position or orientation signals. The outputs of the two modules are integrated with weights that reflect individual estimates of subjective cue reliability, and integrated over time to produce a decision about the perceived dynamics of the input data. Results of this model provided a close fit to the behavioral data, suggesting a mechanism in the human visual system that approximates rational Bayesian inference to integrate position and orientation signals in dynamic form analysis.

## Introduction

The ability to analyze the shape and character of moving objects in the environment is essential for adaptive behavior in a dynamic visual world. Recognizing objects in most real-world settings poses a significant computational challenge to the human visual system due to factors that include signal fragmentation as a result of clutter and occlusion, uncertainty or conflict in sensory information, internal noise in sensory encoding, and ambiguity in the neural representation of an object's shape and its features. Since the world is by no means stationary, human vision must also deal with the fact that objects can undergo changes in shape, viewpoint, and position over time. These changes add yet more complexity and ambiguity to the problem of dynamic form perception. Despite advances on these notoriously difficult issues in work on computational models and video surveillance systems (Aggarwal and Nandhakumar, 1988; Hu et al., 2004), no artificial vision system has approached the inherent capability of human vision in processing and understanding dynamic shapes and images.

In the environment, dynamic form can be broadly categorized as originating from either rigid non-biological shapes with a rigid style of motion (e.g., translating or rotating shapes), or from semi-rigid biological shapes with an articulating style of motion (e.g., human actions or biological motion; see Aggarwal et al., 1998). In the field of biological motion, there are currently two predominant computational approaches to understanding human action perception. One class of models is based on analysis of patterns of local image motion (Webb and Aggarwal, 1982; Giese and Poggio, 2003; Casile and Giese, 2005), while another class of models is based on sequential static form information over time, or dynamic form analysis (Lange and Lappe, 2006; Lange et al., 2006; Theusner et al., 2014). This dichotomy is rooted, in part, in the classical distinction between dorsal and ventral stream processing in the primate visual system (Ungerleider and Mishkin, 1982; Goodale and Milner, 1992). Recent evidence from behavioral (Atkinson et al., 2007; Thurman and Grossman, 2008; Thurman et al., 2010; Thurman and Lu, 2013a), neurophysiological (Vangeneugden et al., 2009, 2011; Singer and Sheinberg, 2010), and functional brain imaging studies (Jastorff and Orban, 2008, 2009; Jastorff et al., 2012; Thompson and Baccus, 2012) is converging on the view that several mechanisms may be employed simultaneously, based on analysis and integration of both motion and form-based features, to support robust action recognition under varying conditions of environmental noise and sensory uncertainty (for review see Blake and Shiffrar, 2007).

Evidence from neurophysiological (Oram and Perrett, 1994; Puce and Perrett, 2003; Vangeneugden et al., 2011) and functional brain imaging studies (Grossman et al., 2000, 2010; Saygin, 2007; Jastorff et al., 2012) further suggest that biological motion may be supported by distinct and specialized neural mechanisms in the human and primate brain. In terms of motion information, behavioral studies suggest the existence of specialized low-level filters for detecting and processing biological actions. For instance, Troje and Westhoff (2006) found evidence for a “life detection” mechanism that is purely motion-based and tuned specifically to characteristic features of terrestrial animals in locomotion (Troje and Westhoff, 2006; Chang and Troje, 2010). Recently, Thurman and Lu (2013b) also found evidence for a basic mechanism that is sensitive to the congruency between the direction of global body motion and the direction implied by intrinsic limb movements, presumably due to the inherent causal relationship between limb movements and whole body movements. Developmental studies have also shown that newborn chicks (Regolin et al., 2000) and human infants (Fox and McDaniel, 1982; Simion et al., 2008) have an innate preference for biological motion, but little sensitivity to biological form information (Vallortigara et al., 2005; Bardi et al., 2010).

Hence, in contrast to motion, it remains unclear whether dynamic form analysis may be specialized for biological actions. From a modeling perspective, the form-based approach to biological motion is computationally analogous on a frame-by-frame basis to generic template-matching schemes employed for rigid shape perception (Liu et al., 1995; Levi et al., 1999). It is highly plausible that this aspect of biological action analysis may actually be supported by a general-purpose system for processing both rigid and non-rigid dynamic objects. Yet, few studies have focused directly on comparing perception of biological to non-biological stimuli (e.g., Neri et al., 1998; Hiris, 2007), particularly in the specific context of form analysis.

The current study was designed to address two specific issues related to dynamic form perception. First, we sought to investigate whether perception of biological stimuli, as compared to rigid non-biological stimuli, is supported by general-purpose or specialized computational mechanisms of form-based visual processing. Secondly, we aimed to examine the relative contribution of two principal cues for visual form—spatial position and orientation—to dynamic form analysis. Previous studies have shown that when position and orientation cues provide conflicting information, they can compete to determine the perceptual appearance of static (Day and Loffler, 2009) and dynamic objects (Thurman and Lu, 2013a). However, the exact nature of the cue integration mechanism remains unclear, as well as the role that sensory uncertainty might play in the cue combination process. In the current study, we created two types of dynamic stimuli (rotating squares and biological motion walkers), and sparsely sampled random positions along the shape of each dynamic stimulus across time (e.g., Beintema and Lappe, 2002), using Gabor patches that provided orientation signals that were either congruent or incongruent with the underlying sampled form. By systematically putting position and orientation information into conflict under varying conditions of sensory uncertainty, we sought to determine the principal rules governing form-based visual cue integration, and to test whether these computational principles apply generally to both biological and non-biological stimuli.

To preview the results, we discovered a characteristic trade-off in the dominance of position and orientation depending jointly on carrier spatial frequency, envelope size, and the number of sampled Gabor elements in the display. Specifically, the appearance of dynamic form was consistent with orientation cues when orientation reliability was high and/or position reliability was low, and vice versa. Importantly, we found no significant differences in the pattern of behavioral results between biological and non-biological stimuli, casting doubt on the notion that biological motion may be specialized in the human brain, at least in specific terms of form analysis. In order to explain individual behavioral data quantitatively, we developed a model of dynamic form analysis using the framework of Bayesian statistics and probabilistic sensory cue integration (for a review see Yuille and Bulthoff, 1996). Bayesian probability theory offers a principled and rigorous method for optimal decision making under conditions with conflicting or uncertain information, and has found support in many aspects of visual perception, including object recognition (Liu et al., 1995), contour integration (Feldman, 2001), motion perception (Weiss and Adelson, 1998), depth perception (Landy et al., 1995), and multisensory cue integration (Ernst and Banks, 2002; Alais and Burr, 2004). Results of our model were highly consistent with individual subject data for both biological and non-biological tasks, supporting the hypothesis of a general-purpose mechanism for dynamic form analysis that integrates orientation and position information in a probabilistic and rational manner according to low-level sensory cue reliability.

## Experiment 1

### Participants

Twenty participants (17 female, mean age = 20.8 ± 3.2 years) were recruited through the Department of Psychology subject pool at the University of California, Los Angeles (UCLA), and were given course credit for participation. All participants had normal or corrected vision, gave informed consent approved by the UCLA Institutional Review Board and were naïve to the purpose and stimuli used in the studies.

### Materials and methods

All stimuli were created using Matlab (MathWorks Inc.) and the Psychophysics Toolbox (Brainard, 1997; Pelli, 1997) and were displayed on a calibrated monitor with a gray background (60 Hz, background luminance 16.2 c/m^2^) powered by a Dell PC running Windows XP. Experiments were conducted in a dark room with a chin rest to maintain a constant viewing distance (35 cm).

The biological motion pattern of human walking was obtained from the Carnegie Mellon Graphics Lab Motion Capture Database, available free online (http://mocap.cs.cmu.edu). Software developed in our laboratory was used to convert the raw motion capture files to point-light format, with 11 points representing the head, mid-shoulder, elbows, wrists, knees, and feet (van Boxtel and Lu, 2013). The horizontal translation component of movement was subtracted so that the animation appeared to walk in place as if on a treadmill, and was trimmed to one walking cycle consisting of 60 frames. The rotating non-biological motion stimulus comprised a sequence of square shape images that were rotated by increments of 6° per frame so that the animation went through one full rotation over the course of 60 frames. Leftward and rightward walkers were created from the same animation sequence by reflecting across the vertical meridian, and clockwise and counter-clockwise rotating square stimuli were created by playing the sequence in either forward or reverse order. Both the biological and non-biological stimuli were presented at a rate of 60 Hz and were equated in vertical size to subtend approximately 9° in height. Stimuli were presented for a duration of 1 s on each trial, so that the biological stimulus completed one full gait cycle (two steps), and the non-biological stimulus completed one full rotation cycle (360°).

**Figure 1:**
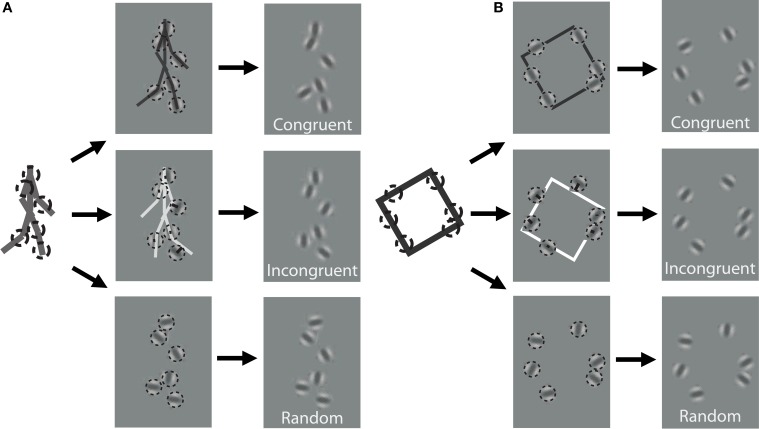
**Schematic of stimulus construction for biological (A) and non-biological (B) stimuli**. The left of each panel shows a single static frame from the animation sequence and an example of 6 random spatial samples denoted by dotted circles. The middle panels show the extraction of orientation from the nearest line segment of the stick figure for a congruent (top) and incongruent (middle) stimulus, as well as a random orientation stimulus (bottom). The right panels show an example of what the stimulus would look like to the observer. Note that the spatial positions of the elements are identical across the three different orientation conditions. Dynamic stimuli in the experiment were created by performing this random sampling procedure independently on each frame of the animation sequence. For video demonstration of incongruent stimuli, see Supplemental Videos 1, 2.

In order to discover the mechanisms underlying dynamic form analysis, we limited the influence of local image motion on perceptual discriminations by creating dynamic stimuli using the one-frame limited-lifetime sampling technique (Beintema and Lappe, 2002; Beintema et al., 2006). By randomly re-sampling a subset of points on every frame of the sequence, local motion information has been shown to be severely disrupted in this type of display, and visual analysis has been argued to proceed on the basis of sequential global form information (Lange and Lappe, 2006). For the biological motion sequence, we first converted the point-light stimuli to a sequence of stick figures by connecting the points according to the anatomy of body structure. The stick figures contained nine limb segments representing the upper and lower arms and legs, as well as the upper torso (shoulders connected to head point). We varied the total number of elements that were randomly sampled per frame depending on stimulus type. For the biological animation sequence, we randomly selected 2, 4, or 6 different limb segments on each frame, and then chose a random position to sample from within the length of each selected limb segment (Figure 1A). For the non-biological motion sequence, we randomly selected 4, 6, or 8 elements from among the four edge segments comprising the rigid square shape on each frame (Figure 1B).

In addition to the locations of the samples, we kept track of the orientation of the limb or edge segment from which each point was sampled, and also calculated the orientation of the nearest limb segment from the corresponding frame of the stimulus with the opposite movement direction. For instance, if the front lower leg was sampled from a rightward walking stimulus on the first frame, then we would extract the orientation of the back lower leg of the leftward walking stimulus on that same frame (Figure 1A). Similarly, for a clockwise rotating animation of non-biological motion, we extracted the orientation of the nearest edge segment on a counter-clockwise rotating stimulus (Figure 1B). Depending on stimulus condition, we manipulated orientation information to be either congruent with the underlying spatially-sampled stimulus, incongruent (i.e., consistent with opposite moving stimulus), or randomized. When randomized, we applied a random offset to the orientation of each element independently, drawn uniformly between 0° and 180°.

In contrast to previous studies that investigated dynamic form analysis in biological motion using broadband positional tokens (e.g., dots; Beintema and Lappe, 2002), we used narrowband Gabor disks that were capable of dually representing both the position and orientation of sampled regions along the shape of each stimulus (Figure 1). Similar stimuli with multiple Gabor elements have been used in previous research to examine global motion perception (Amano et al., 2009; Lee and Lu, 2010) and biological motion perception (Lu, 2010). Gabor patches are well-suited to study these visual processes because they are well-matched to the band pass filtering properties of early visual cortex in terms of spatial frequency, orientation, and spatial scale. All Gabor disks had a fixed phase (sine) and the same suprathreshold level of local contrast (33%). In Experiment 1, the spatial extent of Gabor disks, represented by the standard deviation of the Gaussian envelope, was set to 0.84° visual angle. The only parameter of the Gabor disks besides orientation that changed from trial to trial was the carrier spatial frequency, which allowed us to manipulate the reliability of orientation information on each trial (Burr and Wijesundra, 1991; Day and Loffler, 2009). Decreasing carrier spatial frequency within a small fixed-size envelope (e.g., Gabor) causes a concomitant increase in orientation bandwidth, which has the effect of increasing perceptual uncertainty about the true orientation of the grating (Dakin et al., 1999).

Participants were assigned to one of two groups that reported either the walking direction (leftward, rightward) of biological stimuli (*n* = 10), or the rotation direction (clockwise, counter-clockwise) of non-biological square stimuli (*n* = 10). Subjects indicated their responses with the left and right arrow keys on a keyboard. The experiment had a 3 × 3 × 3 within-subjects design with 3 orientation conditions (congruent, incongruent, random), 3 spatial frequencies (0.25, 0.75, 1.25 cyc/°), and 3 numbers of sampled elements per frame (2, 4, or 6 for biological walkers; 4, 6, or 8 for non-biological rotating squares). Stimulus type (biological vs. non-biological) also served as a between-subjects factor. All trial types were balanced and randomly intermixed in two blocks of 162 trials, resulting in 36 trials per condition and lasting less than 1 h in total duration.

### Results

**Figure 2:**
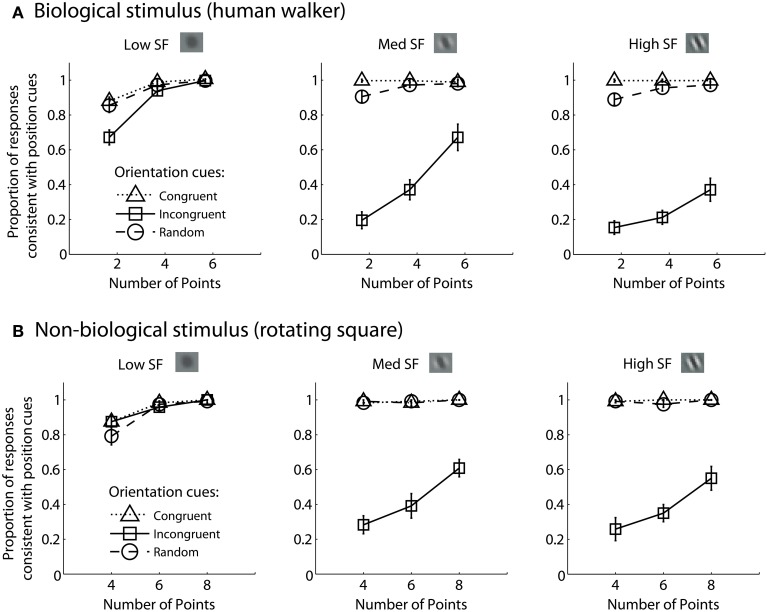
**Mean group data from Experiment 1 for biological (A) and non-biological (B) stimuli**. The panels from left to right show the data from conditions with low, medium, and high spatial frequency elements, respectively. The x-axis represents the three conditions with varying numbers of sampled points per frame. The y-axis shows the proportion of responses that were consistent with the stimulus movement direction defined by the positions of the spatial samples. Values closer to zero in the incongruent cues condition (open squares) indicate perceptual reversals of stimulus movement direction consistent with orientation cues instead of position cues. Error bars represent SEM.

For each experimental condition, we computed the proportion of trials in which observers reported perceiving the stimulus movement direction consistent with position cues. Hence, values closer to zero in the incongruent cues condition would indicate a reversal of appearance away from positional cues and toward the stimulus movement direction defined by orientation cues. Mean group data from the biological and non-biological conditions is displayed in Figure 2. We performed a 3 × 3 × 3 repeated measures ANOVA, with task (biological vs. non-biological) serving as a between-subjects factor. There was a significant main effect of orientation, *F*_(2, 36)_ = 356.2, *p* < 0.001, due primarily to the fact that incongruent orientation caused a significant drop in the proportion of responses consistent with positional cues. Overall, randomizing orientation appeared to have only a small impact on discrimination performance, indicating that observers could effectively ignore noisy orientation cues to perceive the dynamic stimulus on the basis of position cues. Importantly, the strength of the perceptual reversal effect in the incongruent cues condition was modulated by other stimulus parameters. Orientation had a stronger influence on perception as spatial frequency increased, *F*_(2, 36)_ = 138.5, *p* < 0.001, and also had a stronger influence when there were fewer sampled elements in the display, *F*_(2, 36)_ = 236.9, *p* < 0.001. The effect of spatial frequency was likely due to the fact that orientation was more reliable and apparent as spatial frequency increased (Burr and Wijesundra, 1991). The effect of the number of sampled elements suggests that orientation also tended to dominate when there was increased ambiguity and uncertainty about the structure of the underlying stimulus (Day and Loffler, 2009).

Comparing performance in the key incongruent orientation condition between biological and non-biological stimuli, we found that stimulus type was a non-significant between subjects factor, *F*_(1, 18)_ = 3.5, *p* = 0.075. This result indicates that the pattern of performance was not significantly different between the biological and non-biological stimulus conditions. This finding supports the hypothesis that a general-purpose mechanism of dynamic form analysis may underlie performance for both dynamic stimulus types, regardless of the complexity of movement or the biological nature of the visual stimulus. In other words, performance is so well-matched between these two disparate stimulus types in terms of spatial frequency, orientation, and the number of sampled elements, that the simplest and most likely explanation is a common computational mechanism that is not specialized for either biological or non-biological motion.

## Experiment 2

The results of Experiment 1 show that orientation reliability played a critical role in determining the degree to which orientation influenced the perceptual appearance of dynamic biological and non-biological stimuli. Given that position and orientation cues appear to directly compete with each other in this process, we aimed to examine how changes in the reliability of position cues would interact with changes in orientation reliability to influence global dynamic form perception. Previous research has shown that position discrimination performance is more reliable for small as compared to large elements (Toet and Koenderink, 1988), and is independent of carrier spatial frequency (Hess and Holliday, 1992; Day and Loffler, 2009). Hence in Experiment 2, in addition to manipulating spatial frequency across trials, we manipulated the spatial extent of Gabor elements in our displays by varying the standard deviation of the Gaussian envelope. If orientation and position truly compete based on relative cue reliability, we would expect to find systematic trade-offs in the dominance of position and orientation cues as a function of both spatial frequency and Gabor size.

Furthermore, for each participant we quantified the extent to which orientation reliability changed as a function of spatial frequency, and how position reliability changed as a function of Gabor size using two types of low-level feature discrimination task. In the basic orientation discrimination task, participants determined whether a briefly-flashed Gabor disk was rotated either clockwise or counterclockwise from vertical with varying levels of offset in terms of element orientation. In the basic position discrimination task, participants determined whether a briefly-flashed Gabor disk was positioned to the left or right of two flanking Gabor patches located above and below the central test patch with varying levels of offset in terms of element position. We varied spatial frequency and Gabor size across trials and modeled individual subject data with psychometric functions in order to derive empirical estimates of subjective cue reliability.

### Participants

Seven participants (5 female, mean age = 22.6 ± 2.7 years) were recruited through the UCLA Department of Psychology subject pool and given course credit or payment for participation. All participants had normal or corrected vision, gave informed consent approved by the UCLA Institutional Review Board and were naïve to the purpose and stimuli used in the studies.

### Materials and methods

Stimulus construction and display methods were generally the same as in Experiment 1 with a few notable exceptions. Each subject performed two blocks of trials discriminating the walking direction of biological stimuli and two blocks of trials discriminating the rotation direction of non-biological stimuli. All trials presented in these blocks were of the type with incongruent position and orientation cues, while two independent features of the Gabor elements were manipulated. In contrast to Experiment 1 in which Gabor size was fixed, we varied the spatial extent of the Gabor envelope (0.42° or 0.84° SD). In the condition with 0.84° spatial extent, spatial frequency was low, med-low, med-high, and high (0.2, 0.4, 0.8, or 1.6 cyc/°, respectively). In order to equate orientation bandwidth across the two envelope size conditions, we doubled the levels of spatial frequency in the condition with the half-sized (0.42° SD) Gabor envelope (0.4, 0.8, 1.6, or 3.2 cyc/°, respectively). By equating orientation bandwidth, we maintained the reliability of orientation information across the two envelope size conditions (Daugman, 1985).

All Gabor elements had a fixed phase (sine) and a suprathreshold local contrast level of 33%. For each block of trials, envelope size was fixed while spatial frequency varied randomly across trials. The number of elements sampled per frame was also varied randomly across trials depending on stimulus type (2, 4, 6 for walker; 4, 6, 8 for square shape), analogous to Experiment 1. Each block comprised 386 trials, corresponding to 28 trials per condition. The order of completion of conditions was counterbalanced across participants. The experiment had a 4 × 3 × 2 × 2 within-subjects design with 4 spatial frequencies, 3 numbers of sampled elements per frame, 2 Gabor sizes, and 2 stimulus types (biological or non-biological).

In addition, participants performed two types of lower-level feature discrimination tasks. In the orientation discrimination task, two reference Gabor elements with vertical orientation were place 12° above and below a central fixation cross. Gabor elements had a fixed size of 0.84° standard deviation, and spatial frequency was varied across the same four levels as the dynamic shape discrimination tasks (0.2, 0.4, 0.8, or 1.6 cyc/°). On each trial, the fixation cross disappeared and a test Gabor patch was flashed centrally for 17 ms. The test patch had a vertical orientation plus a random offset. The range of offsets varied across eight levels between −20° and 20° depending on the particular spatial frequency condition, in order to sample to entire range of the psychometric function. Participants reported whether the test patch was perceived to be rotated clockwise or counterclockwise relative to the vertical reference patches using the left and right arrow keys on the keyboard. In total, participants completed 768 trials, resulting in 24 trials per condition (4 spatial frequencies and 8 orientation offsets). Cumulative Gaussian functions were used to fit individual subject data, and 1/slope of the psychometric curves served as estimates of orientation cue reliability for each level of spatial frequency.

In the position discrimination task, we varied the standard deviation of the Gabor envelope (0.42° or 0.84°), but fixed spatial frequency (0.4° or 0.8 cyc/°, respectively), across two blocks of trials. Two reference Gabor elements with vertical orientation were place above and below a central fixation cross. The distance between each reference Gabor and the central cross varied depending on size condition (6° or 12°, respectively). On each trial, the fixation cross disappeared and a test Gabor patch was flashed centrally for 17 ms with a random horizontal offset in terms of spatial position. The range of offsets varied across eight levels between −0.67° and 0.67° visual angle. Participants reported whether the test patch was perceived to be located to the left or right of the reference patches using the left and right arrow keys on the keyboard. The orientation of the test patch was orthogonal to the vertical reference patches to avoid the potential use of orientation cues, such as Vernier acuity, for the position discrimination task (Hess and Holliday, 1992). In total, participants completed 512 trials, resulting in 32 trials per condition (2 Gabor sizes, 8 spatial offsets). Cumulative Gaussian functions were fit to individual subject data, and 1/slope of the psychometric curves served as estimates of positional cue reliability for each level of envelope size.

### Results

**Figure 3:**
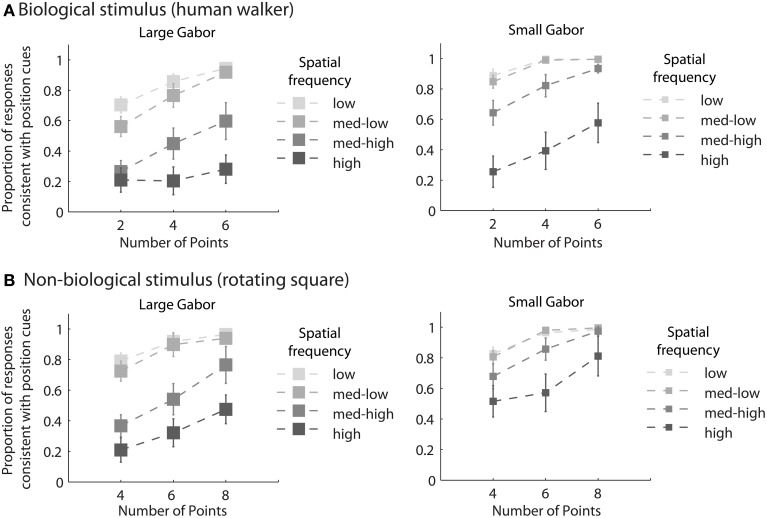
**Mean group behavioral data from Experiment 2 for biological (A) and non-biological (B) stimuli**. The panels from left to right show the data from conditions with larger and smaller elements, respectively, as indicated by the standard deviation of the Gaussian envelope (0.84° or 0.42°). Different spatial frequency conditions are indicated by grayscale squares (see text for SF values in the low, med-low, med-high, and high conditions). The y-axis shows the proportion of responses that were consistent with the stimulus movement direction defined by the positions of the elements. Since all trials were of the type with incongruent orientation and position cues, values closer to zero indicate perceptual reversals of stimulus movement direction consistent with orientation cues. Error bars represent SEM.

Mean group results from Experiment 2 are displayed in Figure 3. A 4 × 3 × 2 × 2 within subjects ANOVA revealed several significant results. Replicating the results of Experiment 1, there was an increasing influence of orientation as spatial frequency increased, *F*_(3, 18)_ = 136.5, *p* < 0.001, as well as an increasing influence of orientation as the number of sampled elements decreased, *F*_(2, 12)_ = 76.5, *p* < 0.001. Also consistent with the results of Experiment 1, stimulus type was a non-significant factor, as performance between the biological and non-biological tasks was very similar, *F*_(1, 6)_ = 3.2, *p* = 0.12. We also found that Gabor size had a significant influence on the relative influence of positional cues, *F*_(1, 6)_ = 79.5, *p* < 0.001. Specifically, there was an increasing influence of positional cues as Gabor size decreased, while (importantly) orientation bandwidth was held constant across the two size conditions. The systematic pattern of changes in the appearance of biological and non-biological dynamic form as a function of both spatial frequency and Gabor size underscores two key points about the global unit formation process in dynamic form analysis. First, information about element position clearly competes with information about element orientation to determine the perceived global stimulus shape. Secondly, changes in the relative influence of position and orientation on behavioral performance appear to be driven by changes in the subjective reliability, or uncertainty, of the low-level visual cues.

**Figure 4:**
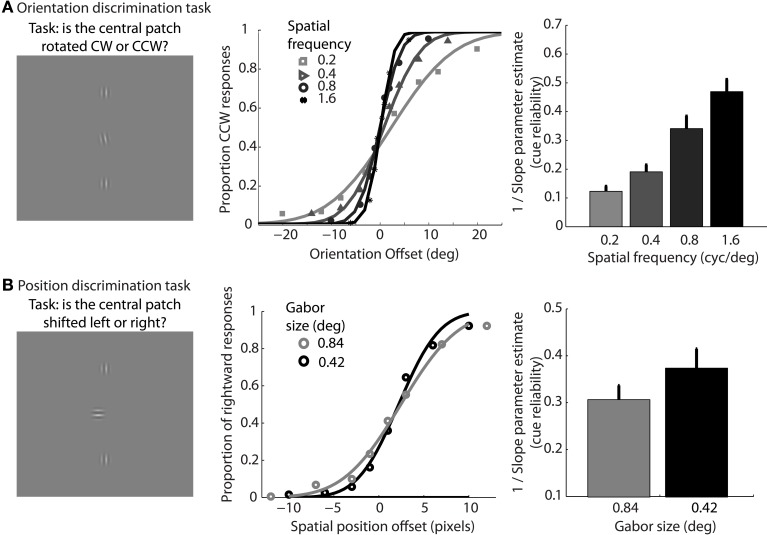
**Mean group data from Experiment 2 for the low-level orientation (A) and position (B) discrimination tasks**. The left panels show example stimuli from the experiment. The middle panels show mean group data for each condition of spatial frequency as a function of orientation offset **(A)**, or each condition of Gabor size as a function of position offset **(B)**. The data are fit with cumulative Gaussian psychometric curves. The right panels show mean group estimates of cue reliability, derived from 1/slope of the psychometric fits. Error bars represent SEM.

In support of this claim, we measured performance in a basic position discrimination task and a basic orientation discrimination task using the same levels of spatial frequency and Gabor size as in the main experiment and within the same group of subjects. Psychometric curves and mean group estimates of cue reliability (1/slope) are displayed in Figure 4. Individual slope estimates for each condition are shown in Table 1. There was a clear monotonic increase in the precision with which observers could discriminate the orientation of a single Gabor disk as a function of carrier spatial frequency (Figure 4A). Similarly, the precision with which observers could discriminate the position of a single Gabor disk increased with decreasing Gabor size (Figure 4B). While these results provide qualitative evidence to support our hypothesis, they also provide empirically-derived quantitative estimates of cue reliability for each subject. Hence, the next goal of the current study was to develop a model of dynamic form analysis in the framework of Bayesian statistics and probabilistic cue combination in order to explain individual subject data on the basis of low-level cue reliability.

**Table 1:** **Overview of slope parameter estimates for each subject from the low-level tasks in Experiment 2**.

**Observer**	**Low SF**	**Med-low SF**	**Med-high SF**	**High SF**	**Large size**	**Small size**
1	8.9	4.45	2.36	1.82	4.41	3.87
2	12.98	8.29	4.95	3.68	4.49	3.84
3	12.22	6.2	3.34	2.27	8.49	4.86
4	11.82	9.59	4.88	2.62	2.45	2.08
5	7.34	4.71	2.2	1.73	2.44	1.63
6	5.59	4.33	3.08	1.75	3.92	3.04
7	5.28	3.57	2.09	2.02	3.73	2.65

The first four columns on the left show slope (σ θ) values associated with orientation discrimination performance for four spatial frequencies (0.2, 0.4, 0.8, 1.6 cyc/°, respectively). The last two columns show slope (σ p) values associated with position discrimination performance for two Gabor sizes (0.84°, 0.42° SD, respectively).

## Model

The present findings pose challenges to both types of current computational models of biological motion, due to the lack of computational mechanisms in these models for explicitly processing both position and orientation information. For example, in the form-based model proposed by Lange and Lappe (2006) and recently updated by Theusner et al. (2014), template-matching is based exclusively on the comparison between observed element positions and stored templates, effectively ignoring the local shape and other characteristics of the elements themselves. This limitation arises from the fact that template-matching is computed through a Euclidian distance metric, comparing the location of perceived elements to locations in stored global body posture templates. Since these models lack an explicit mechanism for processing based on element orientations, as well as an essential pooling mechanism for integrating information about position and orientation, the models would likely fail to predict perceptual reversals for incongruent cues. Moreover, they would certainly fail to account for systematic differences in perception as a function of cue reliability (e.g., changes in spatial frequency and Gabor size).

At the same time, the motion pathway of the computational model developed by Giese and Poggio (2003) would also have difficulty processing the stimuli developed in our study, due to the lack of reliable local image motion information resulting from the sparse random sampling procedure. The form pathway in Giese and Poggio's model does incorporate information about element orientation by virtue of having a dense layer of Gabor filters that serve as the first level of spatial analysis. However, their model lacks a second-order mechanism capable of spatial processing that is invariant to element orientation (e.g., “position label” detector), as well as a mechanism for integrating information based on element position and orientation. In contrast to Lange and Lappe's model, the Giese and Poggio model relies too much on orientation, and predicts perceptual reversals on most trials. We have run preliminary simulations for each of these models in our laboratory using stimuli from the current experiments, and we have confirmed their predictions.

Here we present a computational framework of dynamic form analysis that affords several important advances relative to previous models of biological motion recognition, and that has the capacity to generalize to dynamic form analysis for other non-biological stimuli. The framework is built on two modules for processing different visual cues—position and orientation. The position module performs frame-by-frame template-matching based exclusively on the “position labels” of elements in the display irrespective of orientation. Consequently, processing in this module is similar to the model proposed by Lange and Lappe (2006). The orientation module performs frame-by-frame template-matching on the basis of orientation at each element position. As such, the orientation module is sensitive to both the orientation and relative position of sampled elements in the visual display.

We implemented local Bayesian models for each individual module specialized for position and orientation cues, respectively, and developed an integration operator to combine selections from individual modules with consideration of the reliability of each module. We will first review the local Bayesian models for each module, followed by the integration operator for analyzing the biological motion stimulus, and then discuss how to extend the same model to identify rotation direction of the square stimulus.

**Figure 5:**
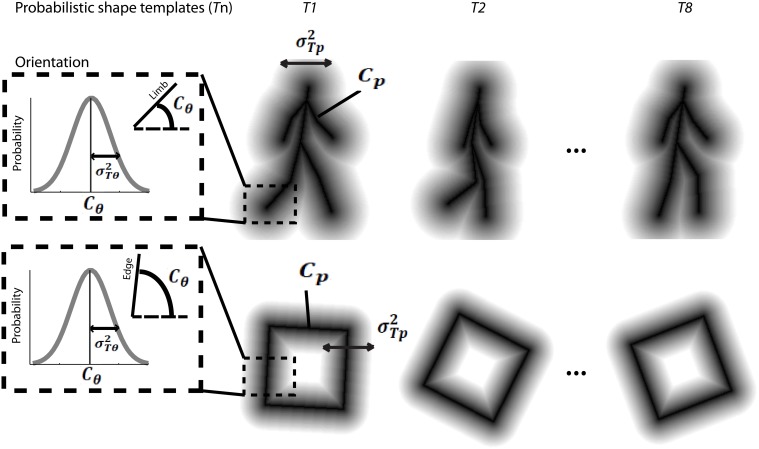
**Schematic depiction of probabilistic shape templates for biological walking stimuli (top), and rotating square stimuli (bottom)**. The dashed square box highlights the probabilistic nature of the templates in terms of orientation information for each edge or limb segment. The mathematical terms are defined in the text.

The model first assumes that the dynamic event sequence is represented as a set of probabilistic shape templates associated with uncertainty. As illustrated in Figure 5, the position templates follow a normal distribution, *T*_*p*_ ~ *N*(*C*_*p*_, σ^2^_Tp_). The means *C*_*p*_ were determined by locations in critical frames, including 8 equidistant frames from the leftward and rightward walking sequences, and 8 equidistant frames from the square rotation sequence, which served as stored templates. The variance σ^2^_Tp_ was determined by the maximum value of closest distances between two neighboring template frames. We found that σ^2^_Tp_ was 8 pixels for walker stimuli, and 13 pixels for rotating square stimuli. The orientation template distributions were defined in a similar way, so that they follow a normal distribution, *T*_θ_ ~ *N*(*C*_θ_, σ^2^_*T*θ_). The means *C*_θ_ indicated the orientation of a given point in the critical template frames, and the variances σ^2^_*T*θ_ were determined by the maximum value of orientation changes of corresponding limbs between neighboring templates. We found that σ^2^_*T*θ_ was 11° for walker stimuli, and 12° for rotating square stimuli. These probabilistic distributions of internal templates reflect the distinctiveness of each presumed template frame, determined by the variability of encoding key postures in biological motion or critical frames in object movements. We tested the model using varying numbers of critical frames in templates between 4 and 16 per animation sequence, and found that model results were robust.

For the position module, 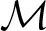
_*p*_, the recognition of the stimulus with sparsely sampled elements is based on the posterior probability of walking direction (i.e., L indicating the left walking direction) conditional on perceived locations ***x***_*p*_. 
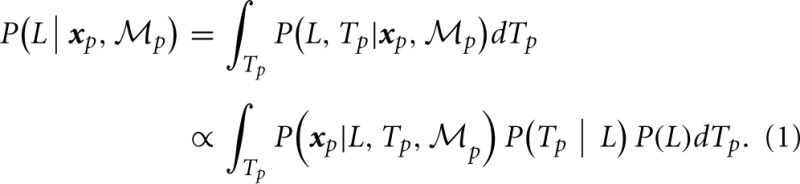


To compute the posterior probability, a deterministic matching between the perceived locations and template locations was assumed to follow a Dirac delta distribution, *P*(***x***_*p*_ | *L*, *T*_*p*_, 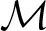
_*p*_) = δ (***x***_*p*_ − *T*_*p*_), with priors on probabilistic templates following a normal distribution with the mean of leftward walker, *T*_*p*_ | *L* ~ *N*(*C*^*L*^_*p*_, σ^2^_*Tp*_) and prior probability of leftward walking direction *P*(*L*) = 0.5. Hence, the probability of determining leftward walking direction by the position module alone can be derived as: 
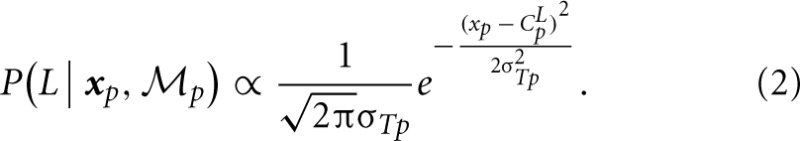


A similar computation can be derived for the orientation module, 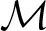
_θ_, based on Bayes rule: 
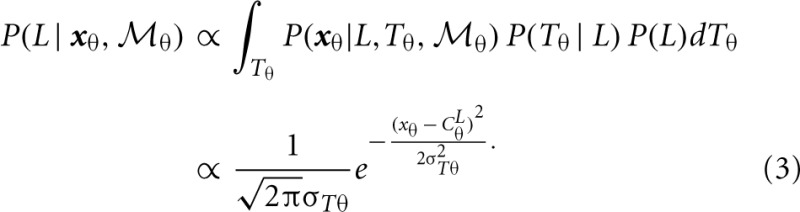


To combine the decisions from the two individual models for form analysis, Bayesian model averaging is used to take into consideration the uncertainty inherent in processing the sensory information within each module. The integrated decision is based on the weighted sum of posterior probabilities calculated from position and orientation modules: 
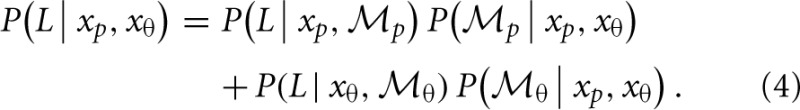


The weights are determined by the sensory noise inherent in individual modules and prior biases to favor position cues relative to orientation cues. Bayes rule is applied to assess the model evidence for each module:w 
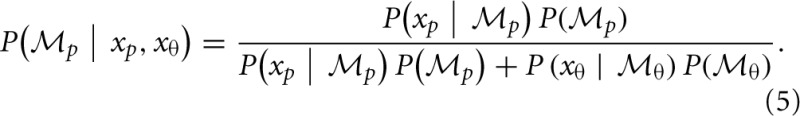


We assume that the variability in perceiving locations and orientation information (i.e., cue reliability) determines the likelihood terms for the two modules as 
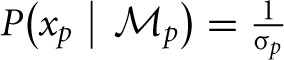
 and 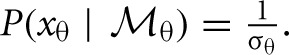. The cue reliability, σ_*p*_ and σ_θ_, can be measured using low-level tasks for each individual subject, as demonstrated in Figure 4 and Table 1. The ratio of prior probability of the two modules is the only free parameter in the model simulation, expressed as 
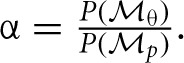
. Hence the integrated decision from the position and orientation modules can be expressed as: 
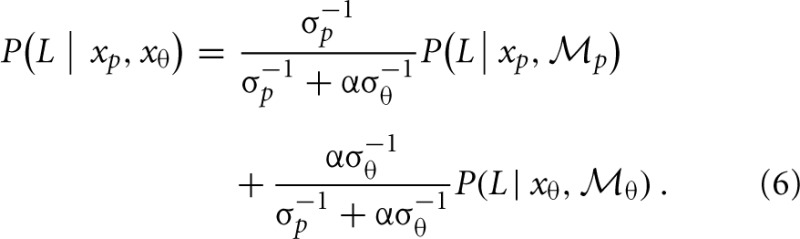


For individual human observers, we used their psychometric performance in the two low-level tasks to measure cue reliability σ_*p*_ and σ_θ_, and then fit the module bias (a single α value) to minimize the discrepancy between model predictions and human performance across 48 experimental conditions.

Since this analysis was performed on a frame-by-frame basis and the stimuli are inherently dynamic, the final stage of the model must integrate the posterior probabilities across time to produce a decision about walking direction. On each stimulus frame we computed the maximum a posteriori (MAP) estimate of the best matching template from among the 8 templates representing each walking direction, and summed the MAP estimates across frames. The decision criterion of the model was to choose the walking direction with the greatest aggregate posterior probability across time.

Finally, applying the same computations to the square rotation task was straightforward with one modification at the decision stage. Because clockwise/counter-clockwise rotating squares involve the same set of template images, but with different temporal orders, the square rotation direction is defined by a specific sequence of frames. Accordingly, the decision stage must implement a mechanism for sequence selectivity during temporal integration. To achieve sequence selectivity, a temporal weighting operation was introduced as follows. First, using the max operator, the model determined the index of the template with the highest posterior probability across all templates within each set of clockwise and counter-clockwise rotating square templates. Next, a sequential matching score was computed by subtracting the max index of the previous frame from the current frame. If sequences are in the correct order, the sequential matching score should be 1 (or close to 1) frame, while deviations from 1 indicate poor sequential matching. Hence, a Gaussian weighting function was used in the form of *W*_*s*_ ~ *N*(1, σ^2^_*s*_), which was centered at 1 frame to penalize sequences that were out of order by giving lower weight to frame sequences that were not ascending consecutively. The sigma of the Gaussian weighting function determined the specificity of sequence selectivity and was estimated to fit group level data (σ^2^_*s*_ = 3.6). To produce a final decision for the square rotation task, the maximum posterior probability from within each set of templates was multiplied by the appropriate weight on each frame and then summed across frames. The model chose the rotation direction with greatest aggregate weighted probability across time.

To model behavioral data for each observer, we ran 100 simulated trials for each experimental condition (48 total conditions). The empirically derived measures of cue reliability, σ_*p*_ and σ_θ_, varied as a function of spatial frequency and Gabor size, and determined the relative weights associated with the orientation and position modules. To estimate the bias parameter, α, for each subject we used least-squares regression. This was the only parameter that was fit to individual subject data, while the relative performance of the model for each condition was determined solely by individual empirical estimates of cue reliability.

### Model results

**Figure 6:**
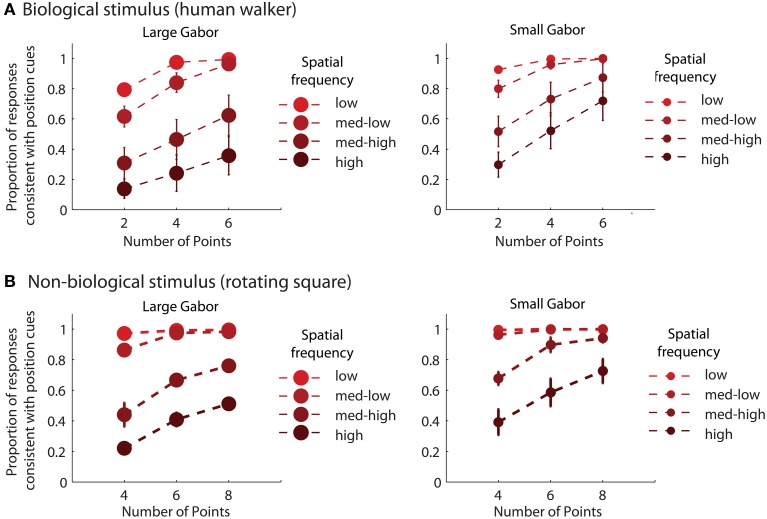
**Group-averaged results of model simulations for biological (A) and non-biological (B) stimuli**. The panels from left to right show the data from conditions with larger and smaller elements, respectively, as indicated by the standard deviation of the Gaussian envelope (0.84° or 0.42°). Different spatial frequency conditions are indicated by colored circles (see Experiment 2: Methods for SF values in the low, med-low, med-high, and high conditions). The y-axis shows the proportion of responses that were consistent with the stimulus movement direction defined by the positions of the elements. Error bars represent SEM.

Table 2 shows the reliability measure (root mean squared errors, RMS) and fitted module bias for 7 participants. Figure 6 shows the group-averaged model results to compare directly with the human data in Figure 3. The model provides good fits to the human data, with an overall correlation across all tasks and observers of *r*_(335)_ = 0.90, *p* < 0.001; *RMS* = 0.095.

**Table 2:** **Overview of the fitted bias parameter and root mean squared errors (RMS) fit between human and model data for each of 7 subjects**.

**Observer**	**Bias parameter (α)**	**RMS biological task**	**RMS non-biological task**
1	5.25	0.120	0.126
2	4.25	0.098	0.104
3	8.0	0.087	0.095
4	8.5	0.064	0.082
5	4.25	0.071	0.076
6	7.75	0.094	0.106
7	6.25	0.143	0.072

## Discussion

The current study documents several significant findings related to dynamic form analysis in the human visual system. First, using the limited lifetime sampling technique to weaken the usefulness of local image motion information and to specifically probe dynamic form processing, we created a novel stimulus in which Gabor elements provided orientation cues that were either congruent or incongruent with information provided by spatial position cues. Importantly, when these cues were put into conflict, we discovered a competitive trade-off in the contribution of position and orientation to perception. This effect appeared to depend strongly on the reliability of visual processing specialized for analyzing the low-level cues. For instance, we found that as spatial frequency increased, orientation cues contributed significantly more to perception of dynamic objects, and that as Gabor size decreased position cues contributed significantly more to perception.

Interestingly, in Experiment 1 we found that random orientation cues yielded minimal impact on discrimination performance, indicating that observers could discount noisy orientation cues that were incompatible with position cues to build a coherent global representation of object shape. At first glance, this result appears to contrast with that of a recent study showing that random orientation cues could impair performance in a task discriminating intact walkers from phase-scrambled walkers (Poljac et al., 2011). We attribute the difference in results primarily to the nature of our task, which was designed to directly assess the perceptual quality and appearance of walking direction as a function of changing orientation cues. In this regard, our approach is similar to that of Day and Loffler (2009), who pointed out that mechanisms for determining the perceptual appearance of an object may differ from mechanisms involved in fine discriminations based on object shape (Loffler, 2008). The key importance of random orientation in the current study was to show that simply violating the consistency between element position and orientation with respect to the global shape (e.g., collinearity) could not explain the significant decrement in performance for the condition with incongruent orientation cues. Hence, the present findings provide strong evidence that changes in performance due to incongruent orientation were caused by genuine reversals in the perceptual appearance of global dynamic shape.

These results contribute to growing evidence that orientation provides a useful and important cue for retrieving information about global structure (Hess and Hayes, 1994; Levi and Klein, 2000; for a review see Loffler, 2008), and extends these findings to perception of dynamic objects (Poljac et al., 2011; Thurman and Lu, 2013a). An important methodological feature of our study was to put position and orientation cues into conflict in order to measure the relative contributions of each type of cue to perception. In fact, Day and Loffler (2009) recently used this approach to study static shape perception by positioning Gabor elements around the edge of a circle and making orientation consistent with the shape of a pentagon. Their results are comparable to ours, showing that orientation was more likely to “capture,” or override, position cues when spatial frequency or Gabor size increased, or when the number of elements in the display decreased. In their discussion, Day and Loffler (2009) reach the same conclusion that we do in the current study, arguing for a global shape processing mechanism that implements weighted cue combination according to sensory cue reliability. Our study goes one step further in developing a Bayesian model of global form analysis and explaining individual subject data using empirical estimates of cue reliability from two low-level discrimination tasks.

Another important aspect of our experimental design was to compare performance between two fundamentally different types of dynamic form. The first task required discrimination of walking direction for biological motion stimuli with semi-rigid form and a complex articulating style of motion. The second task required discrimination of the rotation direction of a rigid square shape with a simpler, rigid style of rotational motion. Despite these differences in the complexity of form and motion information, as well as differences in the biological nature of the stimuli, we found that the pattern of performance in Experiments 1 and 2 was nearly identical between these two tasks and stimulus types. Since the stimuli were designed specifically to exclude local image motion information and to target processes of form analysis, these data suggest that perception of biological and non-biological stimuli on the basis of form cues is likely supported by a common, or generic, computational mechanism. That is, if specialized mechanisms did contribute to biological motion processing, then we would have expected some difference in the pattern of performance across the many variables that we manipulated in the experiments. It is important to note that these findings do not preclude the possibility of specialized mechanisms for biological motion based on characteristic, low-level motion features (Troje and Westhoff, 2006; Chang and Troje, 2010; Van Boxtel and Lu, 2012; Thurman and Lu, 2013b), but they do suggest some independence among processes related to form and motion analysis.

To help explain our behavioral data and to formally test the hypothesis that dynamic form analysis relies on integration according to cue reliability, we developed a Bayesian model that could be applied generically to both biological and non-biological stimuli. The computational principles of the model were inspired by form-based, template-matching approaches to biological motion (Lange and Lappe, 2006; Theusner et al., 2014), and by Bayesian models of optimal, or rational, sensory cue integration (for review see Yuille and Bulthoff, 1996). The model was designed with two assumptions in mind. First, there appear to be two processing pathways for computing global form from displays with sparse local elements. The first pathway assigns position labels to elements in the display and ignores other features, such as element orientation (Levi et al., 1997). Global form perception may be achieved through template matching using internal representations of object shape, on the basis of position cues alone. The second pathway utilizes “snake cues” that are provided by element orientation, and thus considers both orientation and relative position in computing global form (Hess and Hayes, 1994; Levi and Klein, 2000; Day and Loffler, 2009). In order to integrate information from these pathways, a secondary mechanism likely exists to combine their outputs and to produce a single decision on the perceptual appearance of global dynamic form. Our results strongly suggest an integration mechanism that rationally accounts for the relative reliabilities of low-level position and orientation cues; however, it is beyond the scope of the current study to understand exactly how this processing may be implemented in the neural system. These results extend findings from previous studies demonstrating the Bayesian nature of sensory cue integration in other domains of visual processing (Landy et al., 1995; Liu et al., 1995; Weiss and Adelson, 1998; Feldman, 2001), and multisensory processing (Ernst and Banks, 2002; Alais and Burr, 2004).

Form analysis has been recognized as a key component in biological motion perception (Sumi, 1984; Pinto and Shiffrar, 1999; Beintema and Lappe, 2002; Lange and Lappe, 2006; Lu and Liu, 2006; Lu, 2010). Our study provided psychophysical and computational evidence that a generic mechanism of dynamic form analysis can explain perception of both biological and non-biological forms. Within this framework, position, and orientation cues each make independent contributions to the template-matching process and compete to determine the global percept when position and orientation provide conflicting information. The outputs of each computational pathway are later integrated by a mechanism that follows rational Bayesian rules of sensory cue integration, taking into account the relative reliabilities of the low-level cues. Importantly, we found that independent estimates of low-level cue reliability were effective in accurately predicting individual subject performance in two high-level dynamic form discrimination tasks. The good fit between the human behavior and model predictions in the current study provide compelling support for the notion of the “Bayesian brain” (Knill and Pouget, 2004), demonstrating that the visual system uses Bayesian inference in the processing of dynamic form information.

### Conflict of interest statement

The authors declare that the research was conducted in the absence of any commercial or financial relationships that could be construed as a potential conflict of interest.
